# „Left bundle branch (area) pacing“: Sondenpositionierung und Erfolgskriterien – Schritt für Schritt

**DOI:** 10.1007/s00399-024-01060-8

**Published:** 2024-12-02

**Authors:** Joern Schmitt, Till Althoff, Sonia Busch, KR Julian Chun, Tillman Dahme, Micaela Ebert, Heidi Estner, Melanie Gunawardene, Christian Heeger, Leon Iden, Henning Jansen, Victoria Johnson, Tilman Maurer, Andreas Rillig, Sascha Rolf, Philipp Sommer, Daniel Steven, Richard Roland Tilz, David Duncker

**Affiliations:** 1 Westpfalz-Klinikum GmbH, Hellmut-Hartert-Str. 1, 67655 Kaiserslautern, Deutschland; 2 Universitäres Herzzentrum Frankfurt, Frankfurt, Deutschland; 3 Barcelona University Hospital, Barcelona, Spanien; 4 Herz-Neuro-Zentrum Bodensee, Münsterlingen, Deutschland; 5 CCB Frankfurt, Frankfurt, Deutschland; 6 Klinikum Esslingen, Esslingen, Deutschland; 7 Herzzentrum Dresden, Dresden, Deutschland; 8 LMU München, München, Deutschland; 9 Asklepios Klinik St. Georg Hamburg, Hamburg, Deutschland; 10 Asklepios Klinik Altona, Hamburg, Deutschland; 11 Segeberger Kliniken, Bad Segeberg, Deutschland; 12 Elektrophysiologie Bremen, Bremen, Deutschland; 13 Asklepios Klinik Nord-Heidberg, Nord-Heidberg, Deutschland; 14 Universitätsklinikum Hamburg Eppendorf, Hamburg Eppendorf, Deutschland; 15 DRK Kliniken Berlin, Berlin, Deutschland; 16 Herz- und Diabeteszentrum NRW Bad Oyenhausen/Universitätsklinik der Ruhr-Universität Bochum, Bochum, Deutschland; 17 Universitätsklinikum Köln, Köln, Deutschland; 18 Universitätsklinikum Schleswig-Holstein, Lübeck, Deutschland; 19 Medizinische Hochschule Hannover, Hannover, Deutschland

**Keywords:** Physiologische Stimulation, „Left bundle branch area pacing“, „Conduction-system-pacing“, Kardial implantierbare Devices, Schrittmacherinduzierte Kardiomyopathie, Physiological pacing, Left bundle branch area pacing, Conduction system pacing, Cardiac implantable electronic devices, Pacemaker-induced cardiomyopathy

## Abstract

Das „left bundle branch area pacing“ ist die aktuell verbreitetste Form der physiologischen Stimulation vor der His-Bündel-Stimulation. Sie soll die Entstehung einer schrittmacherinduzierten Kardiomyopathie verhindern bzw. beheben und kommt immer häufiger zum Einsatz. Um diese erfolgreich durchführen zu können, bedarf es neben einer Materialkunde v. a. der Kenntnis der spezifischen Anatomie und Röntgenanatomie sowie der EKG-Kriterien einer Linksschenkelstimulation. Dieser Artikel fast die technischen Voraussetzungen und Schritte einer erfolgreichen Implantation zusammen und zeigt Fallstricke auf.

## Abstract (en)

Left bundle branch area pacing is currently the most common form of physiological pacing prior to His bundle pacing. It is intended to prevent or correct the development of pacemaker-induced cardiomyopathy and is being used more and more frequently. In order to be able to perform this successfully, knowledge regarding the specific anatomy and radiological anatomy as well as the ECG criteria for left bundle branch pacing is required in addition to knowledge of the tools. In this article, the technical requirements and steps for successful implantation are summarized and pitfalls are highlighted.

## Einleitung

„Left bundle branch (area) pacing“ (LBBAP) ist eine spezifische Form des „conduction system pacing“ (CSP). CSP beschreibt die Stimulation des Erregungsleitungssystems mittels einer Elektrode im Rahmen der Implantation eines kardialen implantierbaren Devices (CIED). Durch diese physiologische Stimulation soll eine schrittmacherinduzierte Kardiomyopathie verhindert werden, die in etwa bei 4–5 %/Jahr der Patienten bei einer rechtsventrikulären, apikalen Stimulation auftreten kann (> 20 % ventrikulärem Stimulationsanteil; [1, 2]). CSP kann eine Alternative zur klassischen kardialen Resynchronisationstherapie (CRT) darstellen, wenn diese aufgrund anatomischer Gegebenheiten oder elektrischer Limitationen nicht durchführbar ist oder einen primären Ansatz darstellen [3]. Initial erfolgte CSP mittels „His bundle pacing“ (HBP), die Schrittmacherelektrode wird direkt in das His-Bündel eingeschraubt. Hier zeigen sich gute Ergebnisse hinsichtlich des schmalen, physiologischen Stimulationsvektors, jedoch auch einige technische Limitationen, wie höhere Stimulationsreizschwellen und Dislokationsraten. Aktuell ist daher die Stimulation des linken Tawara-Schenkels, bzw. die Stimulation der Region um den linken Schenkel (sog. „left bundle branch area pacing“, LBBAP) in den Fokus gerückt. Unterschieden werden muss, anatomisch, als auch elektrokardiografisch, das selektive und nicht selektive „left bundle branch pacing“ (sLBBP und nsLBBP), das „left fascicular pacing“ (LFP) sowie das „left septal pacing“ (LSP).

In dem nachfolgenden Artikel soll auf die Implantationstechnik, prozedurale Endpunkte und spezifische Komplikationen des LBB(A)P eingegangen und die praktische Durchführung diskutiert werden. Der Zugangsweg zur Sondenplatzierung, das perioperative Management sowie die Implantation einer rechtsventrikulären Schrittmacherelektrode wurden an anderer Stelle beschrieben [4–7].

## Hintergrund

In der aktuell gültigen ESC-Leitlinie zur Indikation der Herzschrittmacherimplantation aus 2021 wurde insbesondere HBP mit einer Klasse-IIb-Empfehlung bei Patienten mit einer LVEF > 40 % und erwartetem Stimulationsanteil > 20 % versehen, IIa lediglich, wenn eine CS-Sondenanlage bei eigentlich bestehender CRT-Indikation nicht möglich ist [8]. Die amerikanische Heart Rhythm Society formulierte in ihrer Guideline eine Klasse-IIa-Empfehlung für ein CSP oder CRT bei allen Patienten mit einem erwarteten ventrikulären Stimulationsanteil > 20 % [9]. In jüngster Vergangenheit folgten Positionspapiere der European Heart Rhythm Association (EHRA) bzw. ein Konsensusdokument zum Einsatz von CSP. In diesen wurde die Bedeutung der physiologischen Stimulation hervorgehoben und gleichsam die rechtsapikale Sondenposition abgewertet [8, 10, 11]. Hier werden Indikationen, aber auch Kriterien für eine erfolgreiche Stimulation des Reizleitungssystems definiert [11] Diese basieren v. a. auf spezifischen EKG-Kriterien. Auch die MELOS-Studie, als größtes europäisches Register mit Daten von 2533 CSP-Implantationen aus 14 erfahrenen europäischen Zentren, spezifiziert den Implantationserfolg an EKG-Kriterien [12]. Auch wenn das Ziel bei CSP ist, mittels Stimulation schmale, physiologische QRS-Komplexe zu erreichen, so sind andere im Verlauf aufgezeigte EKG-Parameter von größerer Bedeutung, ein Cut-off für die „optimale“ QRS-Breite existiert nicht. Im MELOS-Register konnte auch eine größere Erfolgsrate bei Patienten mit normal dimensionierten Herzhöhlen und einer bradykardieassoziierten Indikation erzielt werden, als bei herzinsuffizienten Patienten mit deutlich erweiterten Herzhöhlen [12].

## Vorbereitung zur Implantationsprozedur und Setup im Herzkatheterlabor

Neben den auch sonst üblichen Vorbereitungen sowie der Überprüfung der Indikation und Systemwahl empfiehlt es sich, die EKG-Befunde bzw. wenn vorhanden CT oder MRT besonders zu berücksichtigen. Dies nicht nur im Sinne der Systemwahl, sondern auch um unabhängig von der Erreichbarkeit des LBB ein Ansprechen auf die Therapie antizipieren zu können [13]. Von Bedeutung sind die Diameter des rechten Vorhofs (RA) und des rechten Ventrikels (RV) wegen der Schleusenwahl bzw. -eignung für ein CSP-Verfahren. Dies ist meist von größerer klinischer Relevanz als die Körpergröße des Patienten. Die interventrikuläre Septumdicke spielt eine Rolle, um das Risiko einer Perforation einordnen zu können.

Ein kontinuierlich aufzeichnendes 12-Kanal-EKG ist während der Prozedur die Methode der Wahl und anderen modifizierten EKG-Ableitungen vorzuziehen. Die Anordnung der Defibrillationselektroden sollte die exakte Anordnung der EKG-Elektroden nicht verhindern. Des Weiteren ist es hilfreich, wenn ein 12-Kanal-EKG und die Ableitungen des „patient system analysers“ (PSA) mit den Standardfiltereinstellungen neben den Fluoroskopiebildschirmen simultan gut sichtbar sind.

## Die Prozedur

Die Zugangswege für Schleusen und Sonden werden grundsätzlich analog zu konventionellen Schrittmacherimplantationen, wie von Martens et al. beschrieben, gewählt [6]. Die zu präferierende Präparation der V. cephalica kann ebenso wie eine laterale Punktion der V. subclavia bzw. V. axillaris dazu führen, dass die Schleuse zu kurz ist und das rechts ventrikuläre Septum nicht erreicht wird. Aggraviert wird dies, wenn zusätzlich eine kurze Einführschleuse verwendet wird. Der Standardzugang ist von links, eine Implantation von rechts ist ebenfalls möglich, mit den aktuell verfügbaren Schleusen jedoch meist schwieriger.

**Abb. 1:**
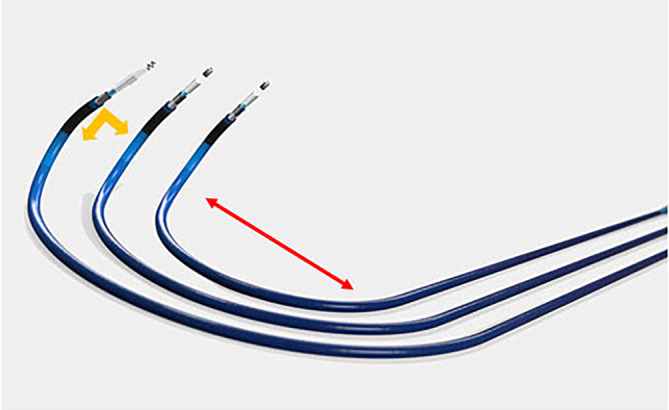
Beispiele von Schleusen mit unterschiedlicher 1. primärer- (Weite, *rot*) und 2. sekundärer-(Angulation, *gelb*) Kurve für die septale Stimulation, das „conduction system pacing“ (CSP)

Die Wahl der geeigneten Schleuse (Abb. 1) aus dem Portfolio des gewählten Herstellers sollte nach einer initialen Fluoroskopie sowie in Kenntnis von Echokardiographie und ggf. CT/MRT getroffen werden. Aktuell bestehen die Portfolios der Hersteller aus bis zu vier nicht-steuerbaren Schleusen, mit je einer distalen (die Ausrichtung zum Septum bestimmenden) und einer proximalen (die Weite/Größe des RA und RV berücksichtigenden) Kurve. Auch steuerbare Schleusen stehen zur Verfügung, mit der Idee einer möglichst großen intraprozeduralen Flexibilität (Abbildung Schleuse). Bei einem normal dimensionierten Herzen ist die kleinste Schleusenkrümmung am ehesten Richtung His-Region gerichtet, die mittlere zieht in Richtung LBBA und die weiteste nach tief septal. Je größer RA und RV sind, desto eher ist die weiteste Kurve geeignet, um die Zielregion für das LBB(A)P zu erreichen. Als Operateur muss man neben der Wahl des Herstellers der Schleuse auch eine Wahl bzgl. der zu verwendenden Elektrode treffen. Es stehen 2 Sondentypen zur Verfügung, zum Einen eine „lumenless lead“ (LLL) ohne Innenlumen, zum Anderen Elektroden mit Innenlumen und Stylet „stylet driven lead“ (SDL). Zu beachten ist, dass aktuell eine größere Erfahrung bei Extraktion mit SDL besteht. Die LLL sind dünner (4,1 F), somit weicher und flexibler, bedürfen allerdings eines Back-up durch die Schleuse und sind ohne diese nicht steuer- und positionierbar. Sie ziehen sich ausschließlich aktiv über die Rotation der Schraube ins Gewebe. Problematisch kann hier eine im Verlauf ggf. notwendige Extraktion werden, da der Einsatz eines „lead locking devices“ zur Sondenstabilisierung und zum Erreichen eines kontinuierlichen Zuges entlang der gesamten Sonde bis zur Spitze nicht möglich ist. Daten und Langzeiterfahrungen und Daten > 10 Jahren liegen nicht vor [5]. Die SDL haben einen Diameter von 5 oder 6F, sind steifer und können zusätzlich zur Schleuse auch über ein in die Sonde eingebrachtes, vorgeformtes Stylet positioniert werden. In der Regel zieht sich die Elektrode über die Rotation der Helix in das Myokard und es ist allenfalls ein sanfter zusätzlicher Druck zum Eindringen notwendig. Fehlt dieser Druck komplett, riskiert man ein sog. „entanglement“, ein Aufdrehen der oberflächlichen Anteile des Septums mit Verletzung und konsekutivem Helixschaden und resultierender nicht ausreichender Fixierung. Sie können bei einer notwendigen Extraktion unter Berücksichtigung der intramyokardialen Lage, wie herkömmlich eingebrachte Sonden behandelt werden. Frühe Daten zeigen einen vergleichbaren Erfolg bei der CSP-Implantation für beide Elektrodentypen und Systeme [14].

**Abb. 2:**
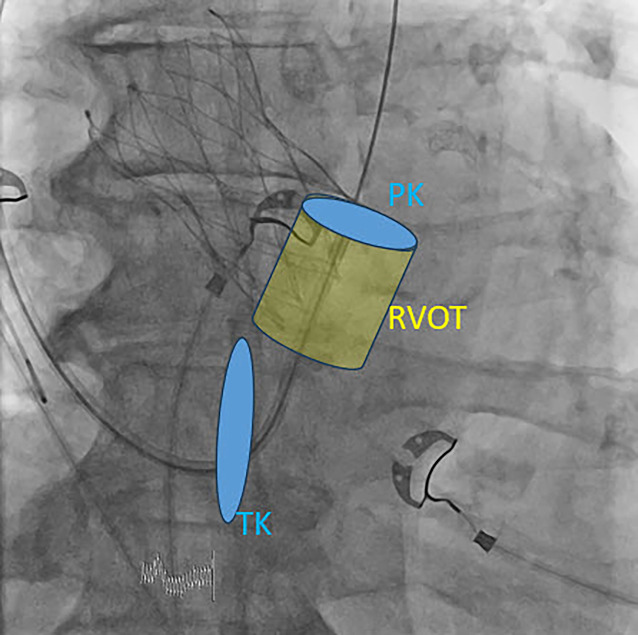
Einbringen der CSP-3D-Schleuse in den RVOT in PA, atraumatisch über einen Führungsdraht. Zur anatomischen Orientierung dient hier eine TAVI-Prothese. *CSP* „conduction system pacing“, *RVOT* rechtsventrikulärer Ausflusstrakt, *PK* Pulmonalklappe, *TK* Trikuspidalklappe, *PA* posterior-anterior

Der Zugang der Schleuse in den rechten Ventrikel sollte atraumatisch über einen langen Schleusendraht mit primärem Ansteuern des RVOT erfolgen (Abb. 2). Dieser Schritt erfolgt typischer weise in AP-Projektion oder in RAO 30°. Erst jetzt sollte die zu implantierende Sonde eingebracht werden.

Ab diesem Schritt ist das Vorgehen je nach verwendetem System/Hersteller und der verwendeten Elektrode unterschiedlich. Bei Sonden, die „stylet driven“ mit aktiv zurückdrehbarer Schraube sind, kann die Schraube vor dem Einbringen in die Schleuse oder nach Ventilpassage in der Schleuse ausgedreht werden. Hier gibt es je nach Hersteller verschiedene Empfehlungen. Wird die Schraube vor dem Einbringen ausgedreht, sollte zum Einbringen das „valve bypass tool“ genutzt werden, um die Schraube nicht am Ventil zu beschädigen. Dies gilt auch für Elektroden mit einer „feststehenden Schraube“, welche von einer löslichen Zuckerkappe geschützt ist. Die hier aufgebrachte Zuckerkappe an der Sondenspitze sollte zuvor aufgelöst/entfernt werden, da dies in der Schleuse nur unzuverlässig erfolgt. Bei dem Ansatz, die Schraube erst nach Einbringen in die Schleuse auszudrehen, muss dies zwingend noch in der Schleuse und sicher komplett erfolgen. Es sollte während des Fixierens der Schraube während des Eindrehens in das Septum darauf geachtet werden, dass die Schraube/Helix nicht retrahiert wird. Einige Hersteller bieten Hilfsmittel zur Fixierung der Helix an, welche eingesetzt werden sollten.

**Abb. 3:**
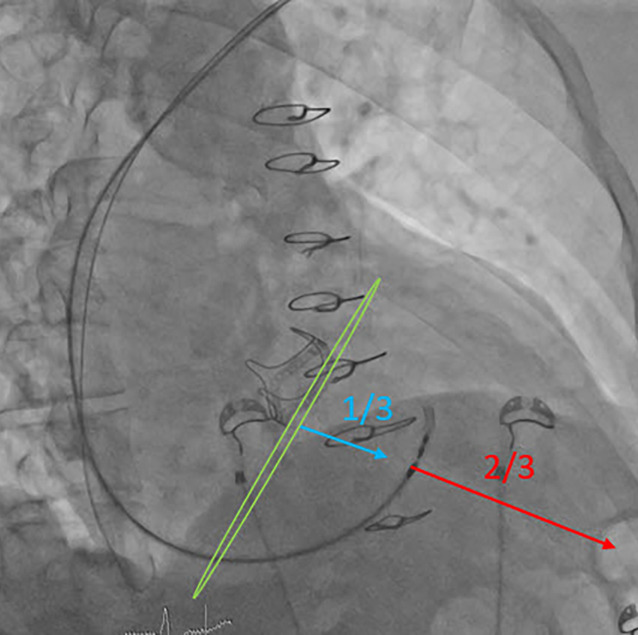
Orientierung in RAO (30°); Abstand von AV-Knoten/Aortenklappe (*blau*) und RV-Apex (*rot*). Die Aortenklappen Prothese markiert die Klappenebene (*grün*) und die AV-Knoten Region (*RAO* „right anterior oblique“, *RV* rechtsventrikulär)

**Abb. 4:**
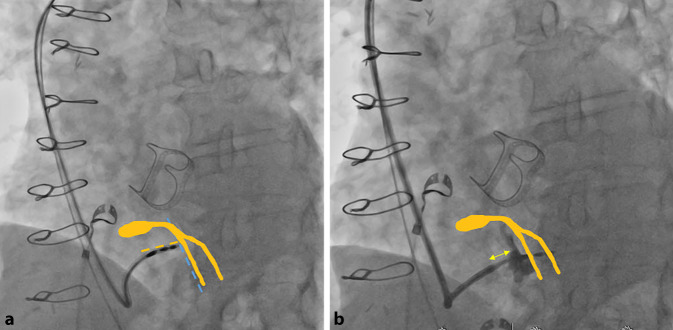
**a** Orthogonales Anstellen der CSP-Schleuse an das Septum (*blau*) in LAO (30–40°) mit anschließendem Einbringen der Elektrode in das Septum. Orientierung am Septum durch Aortenklappen Prothese vereinfacht. **b** Kontrastmittelinjektion nach Rückzug der Schleuse um ca. 1–2 cm mit Dokumentation der Sondenpositionierung tief im Septum (*CSP* „conduction system pacing“, *LAO* „left anterior oblique“)

Nach dem Vorbringen der Elektrode bis an das Ende der Schleuse gilt es, die Schleuse mit der Elektrode an die richtige Stelle am Septum zu bringen und orthogonal auszurichten. Der Rückzug aus dem rechtsventrikulären Ausflusstrakt (RVOT) und die initiale Ausrichtung erfolgen idealerweise in RAO 20–30°, hierbei lassen sich die Position am Septum und Höhe (Basis zu Apex) einordnen (Abb. 3). Um die Klappenebene bzw. das HBE zu annotieren, kann entweder anatomisch/fluoroskopisch vorgegangen werden, oder ggf. auch zusätzlich unterstützend mit einem multipolaren EPU-Katheter das HBE-abgeleitet und fluoroskopisch oder mit einem 3D-Mappingsystem markiert werden. Hilfreich können hier auch zuvor eingebrachte Klappenprothesen, in Aorten- oder Trikuspidalklappenposition sein, welche definierte fluoroskopisch, anatomische Landmarken darstellen. Ist die anatomische Situation unklar, kann auch bereits zu diesem Zeitpunkt eine RV-Angiografie durchgeführt werden, um den RVOT, rechten Ventrikel (RV) und Trikuspidalklappenanulus abgrenzen zu können. Teilt man das Septum von basal nach apikal in 3 Teile wird der Übergang vom 1. zum 2. Drittel angestrebt, analog einer septalen Sondenpositionierung [4]. Typischerweise ist dies 1,5–2 cm vom TK-Ring/His-Bündel in Richtung Apex. Die Schleuse richtet sich in etwa nach 2 Uhr aus. Ist die Position erreicht, erfolgt die Darstellung in LAO 30–40° (Abb. 4). Hier muss neben der orthogonalen Ausrichtung zum Septum auch der Anpressdruck, „Push“, am Septum bestätigt werden, um im Anschluss ein kontrolliertes Eindringen der Elektrode in das Myokard erreichen zu können. Der „Push“ sollte nicht zur Verformung der Schleuse führen, da dies eine Perforation begünstigt. Gegebenenfalls können bereits ventrikuläre Extrasystolen mit septaler Morphologie beobachtet werden und die Lokalisation bestätigen.

Einer der für den Erfolg wichtigsten Schritte der LBBAP-Implantation ist das Einbringen der Sonde in das Septum bis zum Erreichen des Erregungsleitungssystems. Hier muss gleichzeitig auf EKG, PSA und Fluoroskopie geachtet werden, was eine gute, klare Anordnung auf dem Monitor erfordert. Es gilt, die stimulierten Komplexe, die Impedanz (unipolar) bzw. alternativ das sog. Verletzungspotential („current of Injury“, COI, Beispiel siehe [4]) und das Sondenschleusenverhalten im Blick zu haben. Beginnend bei der Sonde muss sichergestellt sein, dass sich die Rotation bis zur Spitze überträgt und die Sonde senkrecht auf das Septum trifft. Je nach verwendeter Schleuse ist es möglich, dass das Ventil die Übertragung blockiert und es zu einem Aufdrehen, „twist“ der Isolation außerhalb der Schleuse kommt. In der Fluoroskopie fehlt dann auch die sonst typische rotierende Helix. Hier kann das Einbringen einer Einführhilfe in die Schleuse das Ventil öffnen und die Sonde freigeben und somit eine bessere Kraftübertragung der Rotation ermöglichen.

**Abb. 5:**
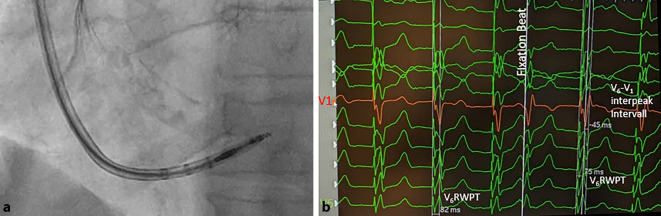
Einschrauben der Elektrode in das Septum mit kontinuierlichem EKG: **a** Fluoroskopisch muss auf eine Rotation der Schraube beim Eindringen geachtet werden.** b** Im EKG zeigt sich eine Verkürzung der „Stimulus‑V_6_ R wave peak time“ (V_6_RWPT) von 82 auf 75 ms, die Entstehung eines R’ in V1 mit ausreichendem V6-V1-Interpeakintervall (45 ms) sowie ein „fixation beat“ der gleichen Morphologie wie die nachfolgend Stimulierten QRS-Komplexe, als Zeichen des Erreichens des linken Schenkels

**Abb. 6:**
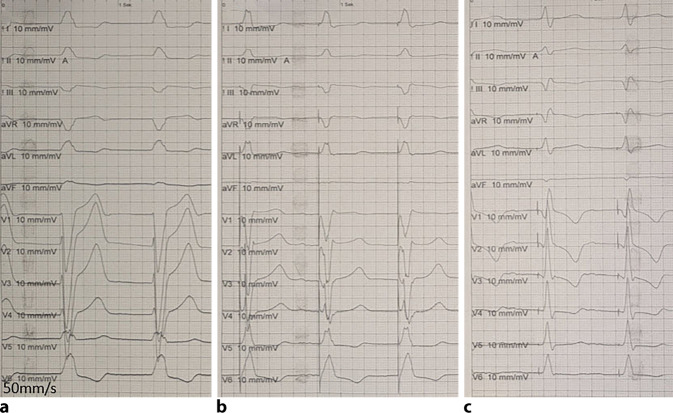
EKG-Beispiel eines Patienten während der Implantation mit nativem Linksschenkelblock (LSB; **a**), nach Anstellen der Schleuse am RV-Septum unter Stimulation (**b**) und letztlich nach Eindrehen der Elektrode in das Septum mit Erreichen eines „left bundle branch captures“ (LBB-Captures) mit „Stimulus‑V_6_ R wave peak time“ (V_6_RWPT) 80 ms bei initialem LSB und nicht mehr vorhandenem Pseudo-Delta in V_6_ ‚sowie R‘ in V_1_ (**c**)

Es gilt die gesamte Elektrode zu drehen und das bis zum Ende vorgebrachte Stylet mit sanftem Druck langsam einzubringen. Wichtig ist, dies unter kontinuierlicher, unipolarer Stimulation durchzuführen (Abb. 5). Die Stimulation dient zum einen dazu, die angestrebte Morphologie des induzierten EKG direkt zu erkennen, zum anderen die Stimulationsimpedanz zu überwachen und eine Perforation bereits früh, bei einem Durchdringen der Schraubenspitze zu bemerken (Impedanzabfall auf < 450 Ω, v. a. ein Delta von > −200 Ω). Auch Veränderungen beim unipolaren Verletzungspotential im Sinne eines plötzlichen Abfalls auf ≤ 5 mV kann hinweisgebend sein, der Verlauf des bipolaren Signals ist dagegen weniger spezifisch [11]. Die minimale bzw. maximale Anzahl an Rotationen lässt sich nicht klar vorhersagen und definieren [15]. In der Regel bedarf es 5–10 kontrollierte Drehungen der Elektrode, um eine tiefe Lage im Septum zu erreichen. Ein Fokus sollte bei Elektroden mit „Retrectable-screw“-Design darauf liegen, den Pin zu arretieren und die Schraube zuvor komplett herauszudrehen. In Abb. 6 ist der intraprozedurale Verlauf des EKG und dessen Wandlung bei initialem Linksschenkelblock (LSB) dargestellt.

**Abb. 7:**
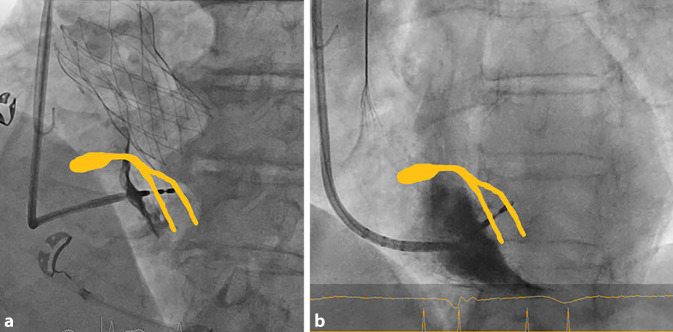
Beispiele einer intraseptalen Sondenlage, Angiografie des rechten Ventrikels (RV) über die 3D-Schleuse: **a** bei einliegender TAVI-Prothese. **b** Ohne weitere anatomische Markierungen. Rückzug der Schleuse sowie gezielte KM-Injektion können helfen, den RV sowie das Septum abzugrenzen. Abbildungen in einer 30° „left anterior oblique“ (LAO 30°)

**Abb. 8:**
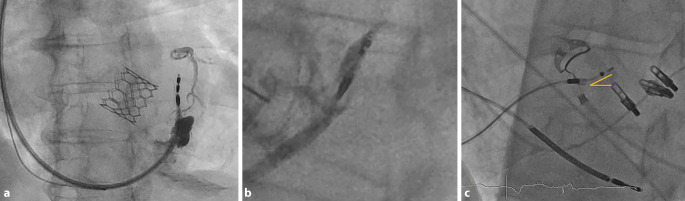
**a** Zu steil in das Septum vorgebrachte Elektrode mit Verletzung eines arteriellen Septalastes und bei Kontrastmittelinjektion der nicht retrahierten und am Septum anstehender Schleuse retrograder Kontrastierung des linken Koronarsystems und intramyokardialem Depot.** b** Kontrastmittelinjektion führt bei anliegender Schleuse und Drilleffekt zur Injektion von KM und die Sonde mit konsekutiver Verschlechterung von Reizschwellen und Sensing sowie Risiko einer 2‑zeitigen Dislokation/Perforation (ähnlich einem Drilleffekt). **c** Abweichen der Elektrode von der Achse der Schleuse mit Verlust des Anpressdruckes, „Push“ und der Steuerbarkeit

Kann trotz tief in das Septum eingebrachter Sonde weder eine selektive noch eine nicht-selektive LBB-Stimulation erreicht werden, sollte eine Umpositionierung erfolgen. Hier ist wichtig zu bedenken, dass jeweils eine Verletzung des septalen Myokards verbleibt. Eine maximal zulässige Anzahl von Umpositionierungen ist nicht definiert, sollte jedoch möglichst geringgehalten werden, nach eigenen Erfahrungen sind max. 3–4 Versuche sinnvoll, um ein optimales Ergebnis zu erzielen. Nach einer EKG-basierten, zufriedenstellenden Positionierung, sollte eine Kontrastmittelinjektion über die ca. 1–2 cm zurückgezogene Schleuse erfolgen. Dadurch kann eine intraseptale Sondenlage verifiziert werden und auch mögliche Fehllagen/Perforationen erkannt werden (Abb. 7). Der Rückzug der Schleuse ist wichtig, um das Kontrastmittel nicht in das Myokard zu injizieren. Dies ist auch nach mehreren Umplatzierungen zur Klärung der anatomischen Situation zu empfehlen. Bei nicht optimalem Einbringen der Sonde in das Septum kann es zu Arrosionen und Verletzungen von koronararteriellen Septalästen kommen, welche sich im Rahmen der Kontrastmittelinjektion anfärben können (Abb. 8). Dies kann asymptomatisch bleiben, aber auch mit ST-Hebungen, Angina pectoris und myokardialen Infarzierungen einhergehen [16]. Die Möglichkeit einer Koronarangiografie mit ggf. Intervention sollte während des Eingriffs bestehen.

Wie bei anderen Implantationen auch kann es intraprozedural zu einem AV-Block III° kommen. Um in Ruhe einen guten Stimulationsort für das angestrebte CSP finden zu können, empfiehlt es sich, die atriale Elektrode temporär zur ventrikulären Back-up-Stimulation einzusetzen und im Verlauf dann im Atrium zu platzieren.

Zum Abschluss der Prozedur wird die 3D-Schleuse analog einer Coronarsinusschleuse geschlitzt. Am sinnvollsten erfolgt das Schlitzen, wenn alle ansonsten zu legenden Sonden positioniert sind, damit es beim Positionieren dieser nicht zu einer Dislokation kommt. Hierzu werden die entsprechenden Schlitzmesser verwendet. Das Stylet sollte nicht bis zum Ende der Sonde eingebracht werden, um mehr Flexibilität für das Ziehen der 3D-Krümmung zu haben.

## EKG-Kriterien: Grundlagen und Definitionen rund um das CSP

Eine korrekte intraprozedurale Anwendung der EKG-Kriterien ist entscheidend für den Erfolg einer CSP-Implantation. Die Ableitung von intrakardialen Signalen über die Sonde, wie z. B. eines Linksschenkelpotentials (über das PSA oder einen Elektrophysiologiemessplatz) hat sich als nicht zuverlässig und verlässlich gezeigt, daher wird die Verwendung der Kriterien des Oberflächen-EKG unter Stimulation empfohlen [11]. Diese Kriterien erfordern die intraprozedurale Ableitung eines 12-Kanal-EKG, mindestens aber eine saubere Ableitung von V_1_ und V_6_. Da dies nicht in allen Implantationsräumen verfügbar ist, wurden Untersuchungen zur Vereinfachung der EKG-Ableitungen gemacht. Dies um eine Implantation auch in Herzkatheterlaboren und OP-Sälen ohne diese Ausstattung zu ermöglichen. Ein PSA-basierter Ansatz nutzt eine modifizierte Anordnung der EKG-Elektroden mit der Idee die essentiellen Ableitungen V_1_ und V_6_ zu imitieren [17]. Da dieses Verfahren dem eines 12-Kanal-EKG nicht gleichwertig ist, sollte es nur in Ausnahmefällen verwendet werden.

Eine Übersicht und Zusammenfassung der am besten untersuchten und evaluierten Kriterien mit Zusammenfassung in einen Algorithmus zur Verifizierung einer LBB-Stimulation beschreibt die EHRA in ihrem Konsensusdokument [11]. Die verfügbaren Kriterien und Cut-off-Werte werden jedoch nicht als absolut und perfekt eingestuft. Der klinische Effekt ist im Vergleich zur His-Bündel-Stimulation und kardialen CRT weniger gut belegt. Im Folgenden sollen für den klinischen Alltag und v. a. während der Implantation gut einsetzbare Kriterien beschrieben werden.

Da wie initial erwähnt intrakardiale Signale nicht ideal sind, sind die folgenden Kriterien unter unipolarer Stimulation zu werten. Es sollte auf einen gut erkenn- und abgrenzbaren Stimulationsspike sowie die Ableitungen V_1_ und V_6_ geachtet werden, und das Ausgangs-EKG gewertet werden (normaler QRS, Rechtsschenkelblock, Linksschenkelblock, Ersatzrhythmus) um im Folgenden die entsprechenden Veränderungen zu erkennen und Erwartungen zu formulieren.

Bereits beim Aufsuchen bzw. Auffinden der geeigneten Position für das Einbringen der Sonde ist das EKG hilfreich. So sollte eine W‑Konfiguration in V1, ein positiver Vektor in II und ein pos./neg. Vektor in III unter Stimulation sichtbar sein.

Am besten geeignet ist 1. Der Übergang und die Veränderung der QRS-Morphologie; 2. Die Zeit von Stimulus zum R‑Wellen Peak („V_6_-R-wave peak time“; V_6_RWPT) und 3. Das V_6_–V_1_ Interpeakintervall (Peak R in V_6_ –> Peak R in V_1)._

1. Der Transition der stimulierten QRS-Komplexe ist v. a. an den R‑Zacken der Ableitungen V_1_ und V_6_ erkennbar (Abb. 5 und 6). Es findet sich die Ausbildung eine r’ oder R’ in V_1_ sowie ein Zuspitzen von V_6_ mit Verlust eines initialen Pseudo-Delta. Dies kann unter Vorbringen der Sonde beobachtet werden oder während eines Reizschwellentests.
2. Neben dem Verlust einer Pseudo-Delta-Welle in V6 kommt es bei Erreichen des linken Schenkels zu einer Verkürzung der V_6_RWPT, dies kann unter Stimulation beim Eindrehen der Sonde sukzessive oder auch sprunghaft geschehen (Abb. 5 und 6). Es ist Ausdruck einer raschen linksventrikulären, homogenen Erregung. Als Grenzwert werden Werte von 75–80 ms bei schmalem Ausgangs QRS oder isoliertem Rechtsschenkelblock definiert und 80–90 ms bei einem Linksschenkelblock, RBB und zusätzlichem faszikulärem Block bzw. Asystolie mit ggf. breitem Ersatzrhythmus.
3. Das V_6_–V_1_-Interpeakintervall bedingt die Ausbildung eines klaren r’ bzw. R’. Es eignet sich v. a. bei initial schmalem QRS-Komplex ohne distinguierte Schenkelblockmorphologie. Anders als die V_6_RWPT verlängert es sich als Ausdruck einer schnellen links und „späteren“ rechtsventrikulären Aktivierung bei Linksbündelstimulation auf ≥ 40 ms, die Grenzwerte liegen hier bei > 40–44 ms [11, 12].

Diese Kriterien, die sich während der Implantation gut beobachten lassen, finden auch in Registerstudien wie z. B. der Melos-Registeranwendung [12].

Ein Phänomen, welches teilweise zu beobachten ist, sind „fixation beats“. Dies sind Extrasystolen, die durch die Irritationen der eindringenden Helix entstehen und beim Erreichen des linken Schenkels ein exaktes Mimikry der oben genannten Kriterien der stimulierten QRS-Komplexe zeigen (Abb. 5).

Die Abb. 5, 6 sowie 9 und 10 zeigen Beispiele eines „conduction system pacing“ und unterschiedlichen Ausgangs-EKG. Die Kriterien werden hier exemplarisch veranschaulicht.

**Abb. 9:**
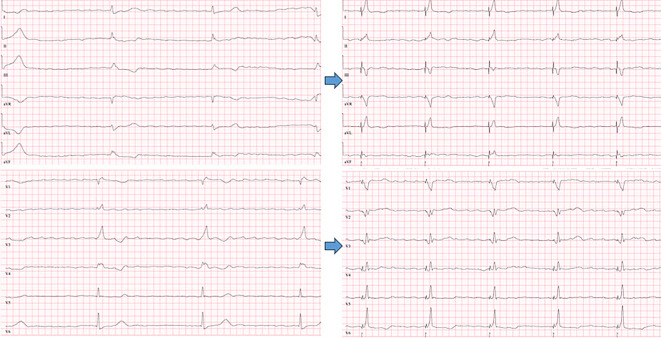
Beispiel vom „conduction system capture“ als links faszikuläres Pacing (LFP) ohne Ausbildung eines terminalen r’/R’ in V_1_, jedoch „V_6_-R-wave peak time“ (V_6_RWPT) < 80ms

**Abb. 10:**
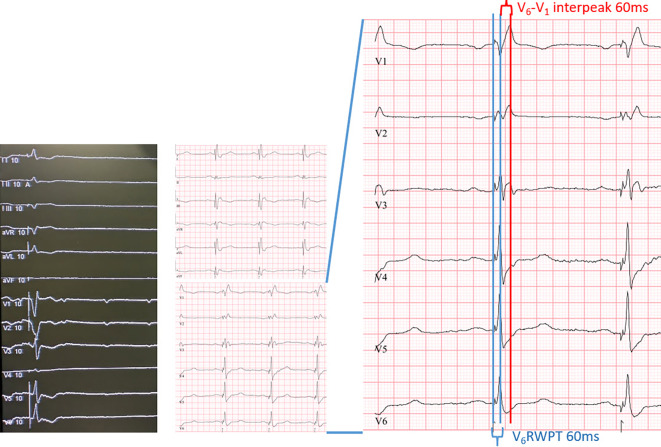
Intraoperatives sowie postoperatives EKG eines Patienten mit AVB III° ohne intraoperativ relevanten ventrikulären Ersatzrhythmus. Die markierten und für ein selektives „left bundle branch pacing“ (LBBP) typischen Leitungszeiten „V_6_-R-wave peak time“ (V_6_RWPT) < 75 ms und „V_6_–V_1_ interpeak time“ > 44 ms zeigen eine links-faszikuläre Stimulation

## Ausblick und Diskussion

Der hier gezeigte Ansatz und die Daten beziehen sich auf Schrittmachersysteme. Es gibt erste Untersuchungen und Daten, welche den Ansatz des CSP und v. a. den des LBBAP auf ICD-Systeme übertragen [18] und damit auch die spannende Diskussionen eröffnet nach der Verwendung von ICD-Aggregaten und Elektroden mit den „alten“ IS1/DF1-Konnektoren, da hier ein Einsatz einer Pace‑/Sense-Elektrode und somit einer wie oben beschriebenen LBBAP oder His-Bündel-Stimulation (HBP) zusätzlich zur konventionellen Positionierung der apikalen ICD-Elektrode möglich wird [19].

Die Ergebnisse prospektiv randomisierter Studien, insbesondere CRT vs. CSP bei verschiedenen Indikationen wie Bradykardie (z. B. AV-Block bei erhaltener Pumpfunktion), aber auch Herzinsuffizienz (LSB und andere Erregungsausbreitungsstörungen bei reduzierter linksventrikulärer Funktion) werden in Zukunft wichtig sein, um eine entsprechende Empfehlung herausgeben zu können. Die aktuell randomisierende „left vs. left“-Studie (NCT05650658) wird mutmaßlich 2029 hilfreiche Ergebnisse liefern können [20].

Ebenso benötigt es in Zukunft vereinfachte und vereinheitlichte EKG-Kriterien, um eine effektive LBB-Stimulation nachweisen zu können und die Prozedur in dieser Hinsicht zu vereinfachen. Die Lernkurve bis zum Erlernen dieser Technik liegt nach eigenen Erfahrungen bei ca. 30–40 Implantationen. Gemessen an den Durchleuchtungszeiten und der Prozedurdauer wird dieser in der Literatur bei 10–20 angegeben [21]. Dies zeigt auch die Komplexität dieser neuen und spannenden Technologie. Auch die stetige Verbesserung der aktuell zur Verfügung stehenden Schleusen wird die Akzeptanz in Zukunft hoffentlich verbessern. Derzeit stehen zumindest im deutschen Abrechnungssystem Vergütungsherausforderungen einem uneingeschränkten Einsatz im Wege.

Ein kürzlich über die EHRA durchgeführtes Survey zeigt die hohe Akzeptanz der Implantationstechnik, da CSP und auch HBP zumindest in Europa trotz eingeschränkter Empfehlungslage bereits in hohem Maße angewendet wird [22]. Als Hürden werden lange Prozedur- und Durchleuchtungszeiten sowie mangelndes Training für die Prozedur angegeben, die ersteren sind sicher durch letzteres behebbar und zeigen sich im klinischen Alltag nach einer Lernkurve als nicht mehr relevant verlängert. Laufende randomisierte, prospektive Studien sowie eine stetige Verbesserung des verfügbaren Materials werden den zukünftigen Einsatz wesentlich beeinflussen und entscheiden, ob das CSP, aber besonders das LBB-Pacing, die Stimulation des Herzens nachhaltig verändern wird.

## Fazit für die Praxis

- Das LBBP („left bundle branch pacing“) wird durch immer besseres Material und mehr klinische Erfahrung einen größeren Stellenwert und eine leichtere Verbreitung erfahren.
- Anpassung des Setup und Vorbereitung sind wichtig. So sollte während der Implantation ein 12-Kanal-EKG zur Verfügung stehen, mindestens jedoch die Ableitungen V1 und V6.
- Für den bestmöglichen Erfolg sollte vor der Implantation eine entsprechende Bildgebung des Septums erfolgen (TTE, CT oder MRT), um die Prozedur optimal planen zu können
- Das LBBP sollte in Zukunft nicht nur bei frustraner CRT-Implantation (kardiale Resynchronisationstherapie) erwogen werden, sondern auch bei Patienten mit zu erwartendem erhöhten ventrikulären Stimulationsbedarf, der Apex als Stimulationsort sollte verlassen werden.

## References

[1] Kiehl EL, Makki T, Kumar R et al (2016) Incidence and predictors of right ventricular pacing-induced cardiomyopathy in patients with complete atrioventricular block and preserved left ventricular systolic function. Heart Rhythm 13:2272–2278. 10.1016/j.hrthm.2016.09.027 27855853 10.1016/j.hrthm.2016.09.027

[2] Abdelmohsen Sayed M, El Fatah Badran AH, Khaled S et al (2022) Stimulation induzierten linksventrikulären Dysfunktion bei Patienten mit Schrittmacher und erhaltener Ejektionsfraktion. Herzschrittmacherther Elektrophysiol 33:312–318. 10.1007/s00399-022-00880-w ((Predictors of right ventricular pacing-induced left ventricular dysfunction in pacemaker recipients with preserved ejection fraction)) 35776180 10.1007/s00399-022-00880-w

[3] Pujol-Lopez M, Jiménez-Arjona R, Garre P et al (2022) Conduction System Pacing vs Biventricular Pacing in Heart Failure and Wide QRS Patients: LEVEL-AT Trial. JACC Clin Electrophysiol 8:1431–1445. 10.1016/j.jacep.2022.08.001 36424012 10.1016/j.jacep.2022.08.001

[4] Wörmann J, Duncker D, Althoff T et al (2024) Elektrodenplatzierung in der kardialen Devicetherapie. Herzschrittmacherther Elektrophysiol. 10.1007/s00399-024-01019-9 ((Lead placement in cardiac implantable electronic devices)) 38748284 10.1007/s00399-024-01019-9 PMC11161426

[5] Reinhardt A, Jansen H, Althoff T et al (2023) Sondenextraktionen bei implantierbaren kardialen Devices. Herzschrittmacherther Elektrophysiol 34:339–350. 10.1007/s00399-023-00963-2 ((Lead extraction in cardiac implantable electronic devices)) 37917360 10.1007/s00399-023-00963-2

[6] Martens E, Sommer P, Johnson V et al (2023) Venöse Zugangswege in der kardialen Devicetherapie. Herzschrittmacherther Elektrophysiol 34:250–255. 10.1007/s00399-023-00954-3 ((Venous access routes for cardiac implantable electronic devices)) 37460626 10.1007/s00399-023-00954-3

[7] Krieger K, Park I, Althoff T et al (2024) Perioperatives Management bei der Versorgung mit aktiven Rhythmusimplantaten. Herzschrittmacherther Elektrophysiol 35:83–90. 10.1007/s00399-023-00989-6 ((Perioperative management for cardiovascular implantable electronic devices)) 38289503 10.1007/s00399-023-00989-6 PMC10879261

[8] Glikson M, Nielsen JC, Kronborg MB et al (2021) 2021 ESC Guidelines on cardiac pacing and cardiac resynchronization therapy. Eur Heart J 42:3427–3520. 10.1093/eurheartj/ehab364 34455430 10.1093/eurheartj/ehab364

[9] Chung MK, Patton KK, Lau C‑P et al (2023) 2023 HRS/APHRS/LAHRS guideline on cardiac physiologic pacing for the avoidance and mitigation of heart failure. Heart Rhythm 20:e17–e91. 10.1016/j.hrthm.2023.03.1538 37283271 10.1016/j.hrthm.2023.03.1538 PMC11062890

[10] Burri H, Starck C, Auricchio A et al (2021) EHRA expert consensus statement and practical guide on optimal implantation technique for conventional pacemakers and implantable cardioverter-defibrillators: endorsed by the Heart Rhythm Society (HRS), the Asia Pacific Heart Rhythm Society (APHRS), and the Latin-American Heart Rhythm Society (LAHRS). Europace 23:983–1008. 10.1093/europace/euaa367 33878762 10.1093/europace/euaa367 PMC12378894

[11] Burri H, Jastrzebski M, Cano Ó et al (2023) EHRA clinical consensus statement on conduction system pacing implantation: endorsed by the Asia Pacific Heart Rhythm Society (APHRS), Canadian Heart Rhythm Society (CHRS), and Latin American Heart Rhythm Society (LAHRS). Europace 25:1208–1236. 10.1093/europace/euad043 37061848 10.1093/europace/euad043 PMC10105878

[12] Jastrzębski M, Kiełbasa G, Cano O et al (2022) Left bundle branch area pacing outcomes: the multicentre European MELOS study. Eur Heart J 43:4161–4173. 10.1093/eurheartj/ehac445 35979843 10.1093/eurheartj/ehac445 PMC9584750

[13] Chen Z, Ma X, Wu S et al (2024) A Comparison of the Association of Septal Scar Burden on Responses to LBBAP-CRT and BVP-CRT. JACC Clin Electrophysiol. 10.1016/j.jacep.2024.03.005 38727661 10.1016/j.jacep.2024.03.005

[14] Sritharan A, Kozhuharov N, Masson N et al (2023) Procedural outcome and follow-up of stylet-driven leads compared with lumenless leads for left bundle branch area pacing. Europace. 10.1093/europace/euad295 37766468 10.1093/europace/euad295 PMC10563653

[15] Chapman D, Morgan F, Tiver KD et al (2024) Assessing Torque Transfer in Conduction System Pacing: Development and Evaluation of an Ex Vivo Model. JACC Clin Electrophysiol 10:306–315. 10.1016/j.jacep.2023.10.035 38206259 10.1016/j.jacep.2023.10.035

[16] Raza MH, Maqbool MU, Kumar S et al (2023) Coronary artery complications with left bundle branch area pacing: A review of literature. Pacing Clin Electrophysiol 46:1222–1229. 10.1111/pace.14822 37708313 10.1111/pace.14822

[17] Su L, Zhu L, Wang S et al (2023) Left Bundle Branch Pacing Facilitated by Novel Surface Electrocardiography in Comparison with Electrophysiology Recording System.

[18] Imnadze G, Fink T, Eitz T et al (2024) Standard Defibrillator Leads for Left Bundle Branch Area Pacing: First-in-Man Experience and Short-Term Follow-Up. JACC Clin Electrophysiol. 10.1016/j.jacep.2024.07.011 39243257 10.1016/j.jacep.2024.07.011

[19] Sticherling C, Ellenbogen KA, Burri H (2024) Stepping back for good reasons: a reappraisal of the DF‑1 connector for defibrillator leads. Europace. 10.1093/europace/euae057 38412340 10.1093/europace/euae057 PMC10919383

[20] Kim JA, Chelu MG (2023) Conduction System Pacing for Cardiac Resynchronization Therapy: The 31-Million-Dollar Question. Tex Heart Inst J. 10.14503/THIJ-23-8118 37308164 10.14503/THIJ-23-8118 PMC10353285

[21] O’Connor M, Shi R, Kramer DB et al (2023) Conduction system pacing learning curve: Left bundle pacing compared to His bundle pacing. Int J Cardiol Heart Vasc 44:101171. 10.1016/j.ijcha.2023.101171 36660200 10.1016/j.ijcha.2023.101171 PMC9843166

[22] Kircanski B, Boveda S, Prinzen F et al (2023) Conduction system pacing in everyday clinical practice: EHRA physician survey. Europace 25:682–687. 10.1093/europace/euac201 36413604 10.1093/europace/euac201 PMC9935001

